# The Role of Secretory Activity Molecules of Visceral Adipocytes in Abdominal Obesity in the Development of Cardiovascular Disease: A Review

**DOI:** 10.3390/biom10030374

**Published:** 2020-02-28

**Authors:** Yuliya I. Ragino, Ekaterina M. Stakhneva, Yana V. Polonskaya, Elena V. Kashtanova

**Affiliations:** Research Institute of Internal and Preventive Medicine - Branch of the Institute of Cytology and Genetics, Siberian Branch of Russian Academy of Sciences, Novosibirsk 630089, Russia; ragino@mail.ru (Y.I.R.); yana-polonskaya@yandex.ru (Y.V.P.); elekastanova@yandex.ru (E.V.K.)

**Keywords:** obesity, adipokines, biomolecules, biomarkers, leptin, resistin, visfatin

## Abstract

Adipose tissue is considered one of the endocrine organs in the body because of its ability to synthesize and release a large number of hormones, cytokines, and growth and vasoactive factors that influence a variety of physiological and pathophysiological processes, such as vascular tone, inflammation, vascular smooth muscle cell migration, endothelial function, and vascular redox state. Moreover, genetic factors substantially contribute to the risk of obesity. Research into the biochemical effects of molecules secreted by visceral adipocytes as well as their molecular genetic characteristics is actively conducted around the world mostly in relation to pathologies of the cardiovascular system, metabolic syndrome, and diabetes mellitus. Adipokines could be developed into biomarkers for diagnosis, prognosis, and therapeutic targets in different diseases. This review describes the relevance of secretory activity molecules of visceral adipocytes in cardiovascular disease associated abdominal obesity.

## 1. Introduction

At present, obesity is a relevant and important problem because of its rapidly increasing prevalence and severity of complications, which sometimes cause death at a young age [1,2].

According to the World Health Organization (WHO) in 2016, more than 1.9 billion adults over the age of 18 were overweight, and more than 650 million of them were obese. The worldwide prevalence of obesity nearly tripled between 1975 and 2016 [3].

Visceral fat constitutes up to 10–20% of all adipose tissue in males and up to 5–8% in females. With age, in people of both sexes, there is an increase in the mass of visceral fat in the human body. Visceral adipose tissue contains a lot of large adipocytes. Visceral adipocytes are metabolically active and possess a higher lipolytic activity [4,5].

In recent years, there have been a number of biochemical and clinical–biochemical studies on the molecules generated by the secretory activity of visceral adipocytes, mostly in relation to the pathologies of the cardiovascular and endocrine systems.

The present review focuses on describing the current state of research in the field of cardiovascular diseases (CVDs) associated with abdominal obesity, discussion of the role of molecules of secretory activity of visceral adipocytes in the development of CVD, and their potential as biomarkers for diagnosis and prognosis.

## 2. Genetic Factors of Obesity

Genetic factors substantially contribute to the risk of obesity [6,7]. In recent years, there have been quite a few molecular genetic studies addressing the obesity problem in the world. It is thought that environmental risk factors related to changes in nutrition and physical activity can materialize only in the presence of genetic factors [8]. Accordingly, identification of candidate genes of obesity has aroused a lot of interest. Through medical examination of children and adults in various populations, investigators have uncovered more than 100 genetic polymorphisms associated with this disease [9,10].

Studies on the genetic polymorphisms in the Alaskan population have shown that the frequency of risk alleles of foodborne diseases in the ethnic groups of these regions is different from that in European and Asian populations [11,12]. A statistically significant association with excess body weight and obesity has been identified for two sequence variants (rs35682 and rs35683) of the adiponectin gene (*ADIPOQ*) in Americans of European origin in contrast to the residents of Alaska, who lack such associations [13]. In the population of Iceland, researchers have discovered a statistically significant association of obesity with polymorphic variant rs7566605 (of gene *INSIG2* regulating the synthesis of cholesterol, phospholipids, triglycerides (TG), and unsaturated fatty acids). This association is absent among the residents of Scandinavian countries, Americans of British origin, Russians living in Siberia, Koreans, and Chinese children and adolescents [14,15].

It is known that during visceral obesity, the increase in adipocyte size is related to the expression of genes FABP4, S100A9, and p53. The expression of p53, but not S100A9, in epicardial stromal cells is associated with adipocyte enlargement in obese patients with CVD [16]. In visceral adipose tissue from patients with IHD (ischemic heart disease), researchers have detected higher gene expression of such cytokines as interleukin-1β (IL-1β), monocytic chemotactic protein-1 (MCP-1), and natriuretic peptide receptor-C (NPR-C) [17]. In a study by Sacks et al., they investigated mRNA expression of 70 genes in visceral adipose tissue from patients with IHD. The authors found that 39 of the 70 analyzed genes were upregulated. Among the most actively expressed mRNAs was *IL8* mRNA, which manifested a threefold increase in expression [18]. It has been reported that circulating tumor necrosis factor-alpha (TNF-α) concentration and its mRNA expression are higher in patients with obesity and IHD (37 subjects) in comparison with patients without cardiovascular disorders (20 subjects). These data confirm the relation between changes in the secretome and transcriptome of adipose tissue in CVDs [19].

According to the above review, a small number of adipokines have been thoroughly studied (adiponectin is among the best-studied adipokines), as is the case for genes involved in the processes of adipogenesis and secretion of a protein product by adipocytes. Nonetheless, in the literature, there are data on some other factors. For instance, in adipocytes of epicardial adipose tissue and subcutaneous adipose tissue, researchers compared the expression of 307 genes participating in the regulation of angiogenesis, formation of vessel morphology, inflammation, and blood clotting. Among the 156 upregulated genes expressed in epicardial adipose tissue, 59 were found to be associated with angiogenesis and inflammation (e.g., *TNFRSF11B, PLAT, TGFB1, THBS2, HIF1A, GATA6*, and *SERPINE1*), whereas among the 166 downregulated genes, only 21 showed such associations (including *ANGPT1, ANGPTL1*, and *VEGFC*). These results indicate that epicardial adipocytes can participate in significant modulation of vascular inflammation [20].

## 3. Specific Molecules of Visceral Adipocytes

Recent studies suggest that visceral adipose tissue not only serves for the accumulation of energy-rich substrates but also acts as an endocrine gland of sorts, which produces various compounds exerting their effects both locally and at the systemic level. Products of secretion by the cells of visceral adipose tissue (adipocytes) are hormones (leptin, adiponectin, and resistin), proinflammatory cytokines (TNF-α, IL-6, IL-8, and others), and rennin–angiotensin system proteins; some of them take part in the functioning of the complement system and in vascular hemostasis (PAI1 and others) [21,22,23,24,25,26]. Research into biochemical effects of the molecules secreted by visceral adipocytes as well as their molecular genetic characteristics is actively conducted worldwide mostly in relation to pathologies of the cardiovascular system, metabolic syndrome, and diabetes mellitus.

## 4. Leptin

Many authors have lately addressed the contribution of leptin to cardiac remodeling in heart failure and the possible explanation of the obesity paradox by the influence of leptin on the metabolism, apoptosis, and remodeling of the extracellular matrix and on hypertrophy. Besides, obesity and hyperleptinemia are often linked with hypertension. We should not rule out the direct action of leptin on such phenomena as atherosclerosis, endothelial dysfunction, and thrombosis [27,28,29]. Unfortunately, extrapolation of the results of basic in vitro research (or research on animal models) to human physiology has turned out to be rather complicated. It is thought that leptin may be an important mediator between obesity and the development of CVDs. This mechanism is possibly driven by such leptin effects as the influence on arterial pressure, aggregation of platelets, formation of arterial thrombosis, and an inflammatory vascular response. Investigators believe that a high level of leptin is related to low arterial extensibility and participates in the pathogenesis of atherosclerosis via mechanisms different from vascular relaxation. Some authors have noted a connection between serum concentrations of leptin and various cardiovascular risks, including stroke, chronic heart failure, acute myocardial infarction, coronary artery disease, and left ventricle hypertrophy [30,31,32,33,34]. It seems worthwhile to study leptin/adiponectin ratios. As demonstrated by Kappelle et al. [35] in a cohort case–control study of males with adjustment for age, the incidence of CVDs correlates with blood plasma levels of leptin, adiponectin, and the leptin/adiponectin ratio. After adjustment of the results for smoking status, waist circumference, hypertension, microalbuminuria, the TG/high-density lipoprotein (HDL) ratio, C-reactive protein (CRP), and the homeostasis model assessment of insulin resistance (HOMA-IR) index, the statistical significance of the link between CVD incidence and leptin and adiponectin levels disappeared. Thus, the authors concluded that the leptin/adiponectin ratio may be the most sensitive marker of CVD in males in comparison with leptin and adiponectin.

## 5. Adiponectin

In contrast to most other adipokines, the blood plasma level of adiponectin is lower during obesity and during the related pathologies, including CVDs, T2DM, and nonalcoholic fatty liver disease (NAFLD) [36,37,38]. Adiponectin levels were shown to be significantly lower in patients with NAFLD and NAFLD + T2DM than in the group of healthy subjects. Lower level of adiponectin was associated with the presence of T2DM and NAFLD independent of insulin resistance and obesity indices [37]. The number of studies on the relationship between NAFLD and CVD is increasing year by year. These studies suggest that NAFLD can be actively involved in the pathogenesis of CVD [37,39].

Both mRNA expression of adiponectin and secretion of oligomeric adiponectin of high molecular weight are dysregulated in the adipose tissue of obese people. Epidemiological studies in various ethnic groups indicate that a low level of adiponectin in blood serum, and especially of its high-molecular-weight oligomer, is an independent risk factor of CVDs. There is a strong correlation between hypoadiponectinemia and CHD. Conversely, high blood plasma levels of adiponectin are related to a lower CHD risk, independently of other risk factors [40]. Hypoadiponectinemia is independently associated with endothelial dysfunction in patients with diabetes. The inverse correlation between intima/media thickness and serum adiponectin has been detected in several clinical cohorts, which included both healthy subjects and patients with diabetes mellitus of both sexes. Moreover, the ratio of leptin to adiponectin is inversely related to the intima/media thickness of the vascular wall [41] and has been suggested as an atherosclerotic indicator in patients with T2DM. Hyperadiponectinemia is an independent risk factor of diabetic cardiomyopathy. In healthy people, the levels of total adiponectin and high-molecular-weight adiponectin in the blood are linked with left ventricle hypertrophy, regardless of age and metabolic factors [42]. A similar association has been noted in obese people [43]. These paradoxical findings can be explained by the development of adiponectin resistance [44] with age and with CVD progression.

## 6. Resistin

In 2001, researchers isolated a polypeptide named resistin, which is secreted mostly by preadipocytes and, to a lesser extent, by mature adipocytes located mostly in the abdomen [44,45]. There is evidence that CVDs are accompanied by changes in serum levels of resistin [46,47,48]. For instance, one study included 220 patients with chest pain and revealed that in patients with acute coronary syndrome (ACS), serum levels of resistin were significantly higher than those in the patients with stable angina pectoris. In the ACS group, an elevated serum level of resistin significantly correlated with high-sensitivity serum C-reactive protein (CRP) test results and with the leukocyte count; resistin also correlated with the number of coronary vessels with stenosis >50%. Overall, serum resistin was found to be a strong risk factor of ACS [49]. Moreover, resistin is a prognostic factor for death in type 2 diabetes [50].

There is a report of a substantial increase in the blood plasma level of resistin in patients with unstable angina pectoris in comparison to patients with stable angina pectoris or a control group; again, plasma resistin positively correlated with indicators of inflammation and of endothelium activation, e.g., the leukocyte count, CRP, and blood level of endothelin 1. Furthermore, resistin was found to be an independent predictor of the main adverse cardiovascular events, including cardiovascular death, myocardial infarction, and restenosis in patients after transcutaneous transluminal coronary angioplasty [51,52]. In a European cohort study on cancer and nutrition (the Potsdam study on 26,490 middle-aged patients without a history of acute myocardial infarction (AMI) or stroke; showing a relative risk of 2.09), the authors proposed to investigate the blood plasma level of resistin (adjusted for CRP) to predict the development of AMI. They noted elevated levels of resistin in patients with ACS and its correlation with severe myocardial injury and poor prognosis. A high level of resistin apparently is related to an adverse outcome after atherothrombotic ischemic stroke, regardless of other predictors of adverse outcomes [53]. Nevertheless, in some studies, researchers did not find a link between an elevated level of resistin circulating in the blood and the prevalence or outcome of IHD. Such discrepancies may have to do with differences in the demographics of the studied groups, differences in study design and in eligibility criteria for participants, as well as dissimilar methods of analysis. All these observations suggest that resistin plays an important part in the pathogenesis of CVDs, and currently, studies are underway to determine its involvement in atherogenesis and ACS.

## 7. Tumor Necrosis Factor Alpha (TNF-α)

Tumor necrosis factor-α is expressed by lymphocytes and adipocytes and has auto- and paracrine effects. The level of TNF-α in adipose tissue correlates with fat mass and hyperinsulinemia. The TNF-α stimulates leptin secretion, and this action is mediated by IL-1. In animal experiments, TNF-α administration reduces food intake, delays gastric emptying, inhibits insulin effects, modulates the levels of glucagon and glucocorticoids, and stimulates thermogenesis. Because of elevated secretion of TNF-α and IL-6, visceral fat exerts a proinflammatory action. These proinflammatory cytokines activate a transcription factor of the response to oxidative stress, NF-κB. The vascular endothelium is a specific target of TNF-α: the latter upregulates many proinflammatory, procoagulant, pro-proliferative, and proapoptotic genes [54,55]. The common initial stage for these changes is a decrease in the bioavailability of nitric oxide (NO), which is secondary to increased elimination of NO by reactive oxygen species (ROS) and/or decreased synthesis of NO. Vascular damage related to the main risk factors of CVDs [56] is characterized by endothelial dysfunction, formation and liberation of inflammatory cytokines such as TNF-α, prolonged activation of the systems producing ROS, and eventually, a decrease in endothelial bioavailability of NO. In isolated coronary arteries of type 2 diabetic mice, it was demonstrated that activated superoxide-generating systems reduced vasodilatation, and the concentration of circulating TNF-α increased. Inhibition of superoxide production [57] or lowering of superoxide content restores vasodilatation. In aged rats, there is an increase in the blood level of TNF-α, which is associated with endothelial dysfunction of coronary arteries, whereas chronic inhibition of TNF-α improves the slowing of blood flow in mesenteric arteries of the aged rats. Thus, TNF-α attenuates vasodilatation, mostly by lowering NO bioavailability and also because of ROS upregulation.

## 8. Interleukin-1 (IL-1)

Interleukin-1 is an apical proinflammatory mediator during acute and chronic inflammation and is a powerful inducer of an innate immune response [58]. It triggers the synthesis and expression of several hundred mediators of the secondary alteration and upregulates its own production and processing: this stage is a key event in the pathogenesis of many autoinflammatory diseases [59,60,61]. Interleukin-1α and IL-1β, together with their negative regulator IL-1 receptor antagonist, play the most important role in the development of various CVDs, including atherosclerosis, AMI, myocarditis, dilated cardiomyopathy, infectious endocarditis, and cardiomyopathy. All other variants of the IL1 gene also correlate with a higher risk of CVDs. Two related genes encode two different proteins (IL-1α and IL-1β), which bind to the same receptor (type I). Interleukin-1α is synthesized as a fully active peptide, which remains bound to the membrane or may be liberated from the cytoplasm during cell death. Consequently, IL-1α participates more prominently in a local response to injury, and less frequently in a systemic inflammatory response [59,60,62]. Interleukin-1β, the main form of circulating IL-1, is initially synthesized as a precursor (pro-IL-1β) and becomes active via cleavage of pro-IL-1β by caspase 1 under the conditions of a macromolecular structure known as a focus of inflammation [59]. Caspase 1 also participates in the secretion of active IL-1, which can then bind to a membrane receptor of IL-1 on the same cell, on a neighboring cell, or on target cells [60]. Inflammation activation after tissue injury causes a local increase in the IL-1β amount, thereby substantially enhancing the inflammatory response, attracting a large number of inflammatory cells, stimulating metalloproteinases, and eventually inducing the death (pyroptosis) of such inflammatory cells as leukocytes and resident cells [63,64]. In other words, the active form of IL-1 is a consequence of transformation of an inactive precursor through the NLRP3/caspase pathway after an infectious or inflammatory stimulus. Interleukin-1β possesses various biological properties and correlates with atherosclerosis, IHD, and tissue remodeling after AMI. IL-1α is constitutively active toward various cell types by binding to the same receptor as IL-1β binds, thereby exerting similar actions. Physiologically, IL-1α acts as a danger signal in response to sterile irritants, mostly owing to cell death as a consequence of necrosis taking place in AMI or stroke.

## 9. Interleukin-6 (IL-6)

Interleukin-6 is produced by activated monocytes or macrophages, by a vascular endothelium, fibroblasts, activated T cells, and by several nonimmune cell types [65]. Interleukin-6 performs its biological functions via two signaling pathways: classical signaling through membrane-bound receptor of IL-6 (IL-6-R), which is responsible for anti-inflammatory processes, and signal transduction via a soluble receptor of IL-6 (sIL-6-R), which takes part in proinflammatory processes. Some effects caused by IL-6 are similar to those seen during the actions of IL-1 and TNF. Nonetheless, the main effect of IL-6 is related to its participation as a cofactor in the differentiation of B lymphocytes, their maturation, and conversion to plasma cells secreting immunoglobulins. In addition, IL-6 promotes IL-2 receptor expression on activated immune cells and induces IL-2 production by T cells. This cytokine stimulates T-lymphocyte proliferation and hematopoietic reactions. Furthermore, IL-6 mediates acute and chronic inflammatory reactions and regulates the acute phase reaction in hepatocytes, including the synthesis of CRP [66]. Vascular effects of IL-6 depend on experimental conditions. In C57B1/6 mice and in apoE-deficient mice, injection of recombinant IL-6 at supraphysiological doses enhances atherosclerosis. By contrast, at an early stage, deletion of the IL6 gene in apoE-deficient mice did not affect the disease relative to control mice, whereas at later stages, this knockout promoted atherosclerosis. Atherosclerotic plaques of humans contain IL-6, and a high plasma level of IL-6 is associated with a poor prognosis in people without a well-pronounced CVD and in patients with ACS. A large-scale analysis of 34 genetic studies, including 25,458 cases of IHD and 100,740 control cases of polymorphisms of the IL-6-R gene with cardiac events, revealed that polymorphism rs7529229 in IL6R, which is associated with elevated blood plasma levels of soluble IL-6-R, and lower levels of CRP correlate with lesser frequency of coronary artery diseases [67]. The similar findings of the two independent genetic studies indicate a causal link between IL-6 signaling and atherosclerosis in humans. Therefore, IL-6-R is a promising therapeutic target in IHD.

## 10. Interleukin-8 (IL-8)

Interleukin-8 bears the main responsibility for chemotaxis (recruiting) of monocytes and neutrophils: the characteristic cells of an acute inflammatory response. In vivo, a chemotactic gradient can be created by the binding of IL-8 to proteins of the basal membrane. This gradient helps to lead cells to an inflammation site and retains them as soon as they enter the site. Aside from recruitment of cells, IL-8 promotes activation of monocytes and neutrophils. Interleukin-8 emerges at an early stage of an inflammatory response but stays active for a long period: several days or even weeks. This feature distinguishes it from other inflammatory cytokines, which are usually produced and metabolized in vivo within several hours. IL-8 is very sensitive to oxidative agents, whereas antioxidants substantially downregulate the IL8 gene. The role of oxidative agents in the regulation of IL-8 and other chemokines is important in the pathogenesis of CVDs, where ischemia-induced oxidative stress is simultaneously a marker of the disease and a potential therapeutic target [68]. In the literature, there are plenty of data on the involvement of IL-8 in the pathogenesis of atherosclerosis. A popular field of research is elucidating whether IL-8 is a predictor of short-term and long-term outcomes in patients with IHD. Inoue et al. has found serum levels of 10 cytokines to be prognostically significant in an analysis of long-term outcomes of patients with stable IHD confirmed by angiography. The authors concluded that IL-8 is the only cytokine that predicts cardiovascular complications and does so regardless of nine other cytokines and high-sensitivity CRP test results [69].

It has been proven that IL-8 is a strong independent prognostic factor of cardiovascular and general mortality among patients with end-stage kidney failure. Investigators have found that the initial concentrations of IL-8 are substantially higher in IHD than in patients without IHD. Nonetheless, adjustment for additional cardiovascular and immunological risk factors weakened the observed relation, and the authors concluded that elevated blood levels of IL-8 precede the development of IHD but cannot serve as an independent risk factor. It is reported that lower levels of IL-8 after cardiovascular interventions are a prognostic indicator of more favorable short-term and long-term outcomes of the disease [70].

## 11. Interleukin-10 (IL-10)

Interleukin-10 controls the level of active forms of cyclooxygenase (COX). Sikka et al. has revealed that IL-10 deficiency causes COX activation and, therefore, activation of thromboxane receptor, which induces endothelial and cardiac dysfunction of blood vessels in mice. In mice with a low level of IL-10, cardiac and vascular dysfunctions develop with age [71]. On the other hand, Didion et al. has demonstrated that endogenous IL-10 reduces angiotensin II-mediated oxidative stress and vascular dysfunction both in vitro and in vivo, thereby confirming that at least some protective functions of IL-10 can be performed in the vessel wall [72]. Elevated initial levels of IL-10 are an independent predictor of a higher risk of subsequent death and AMI within one year. This finding is confirmed by the study by Cavusoglu and coauthors, who discovered that elevated levels of IL-10 in plasma are independently associated with a higher risk of death and of nonlethal myocardial infarction during 5-year follow-up in a group of males with ACS who were referred to coronary angiography. Additionally, the prognostic value of IL-10 in this regard did not depend on other biomarkers, including high-sensitivity CRP test results, and was comparable with their prognostic utility [73]. Elevated levels of IL-10 have also been found in patients with acute myocarditis, after which IL-10 was suggested as a pathogenic marker that can help to discriminate acute myocarditis and AMI [74]. Elevated levels of IL-10 in blood serum have also been associated with a higher incidence of subsequent adverse events in patients with cardiomyopathy [75]. Thus, IL-10, originally known as an anti-inflammatory cytokine with pleiotropic effects on the human body, probably plays an ambiguous role in CVDs.

## 12. Tissue Factor (TF)

As a strong initiator of coagulation, tissue factor (TF) is a crucial player in hemostasis and thrombogenesis. In general, TF is absent in the cells of a vascular endothelium, or if it is present, its conformation does not allow it to interact with factor VII. Nonetheless, after tissue and vascular endothelium are damaged, and the physical barrier separating intravessel factor VII from TF is disrupted, the factor VII–TF complex is formed. Furthermore, during stimulation of monocytes, macrophages, and endothelial cells by endotoxins, cytokines, and lectins, the expression of TF increases in these cells in parallel with an increase in procoagulant activity [76]. In ACS, concentrations of inflammatory cytokines, such as TNF-α and interleukins, increase at the site of coronary artery occlusion to such an extent that TF is produced in vascular cells. There are several polymorphisms of the TF gene, and existing evidence suggests that certain mutations in gene TF and in the promoter of this gene may be related to a worse outcome of patients with ACS, possibly because of elevated production of TF by monocytes [77]. In patients with tension-related unstable angina pectoris or myocardial infarction without elevation of the ST segment and a high index of intima/media thickness (TIMI ≥ 4), an elevated level of TF is detectable in blood plasma in comparison to patients with a low TIMI index (<3). Elevated levels of TF are present in people with cardiovascular risk factors and in patients with IHD. Tissue factor expression is higher in atherosclerotic plaques, and cellular and extracellular TF (contained in microparticles) come into contact with blood during endothelium erosion or plaque rupture. Consequently, TF plays a decisive part in the development of acute vascular events, such as AMI or stroke. On the other hand, TF can promote atherosclerosis progression by enhancing migration and proliferation of smooth muscle cells in the vessels.

## 13. Lipoportein Lipase (LPL)

In a large cross-sectional study by Khera et al., they found that the presence of rare damaging mutations in the lipoprotein lipase gene (LPL) was substantially associated with higher levels of TGs and a diagnosis of IHD [78]. Similar results have been obtained by Lotta et al., who uncovered a relation between a polymorphism of the LPL gene and a lower risk of IHD among people carrying alleles corresponding to low TG levels in the blood, irrespective of genetic mechanisms lowering the level of low-density lipoprotein (LDL) cholesterol [79]. In international literature, rather frequently, there are descriptions of both isolated cases and of meta-analyses suggesting that low levels of LPL in serum are linked with early atherosclerosis (before 55 years of age), whereas a higher LPL activity has a protective effect against the development of IHD. Xie and Li in a meta-analysis show that the risk of IHD varies depending on the polymorphism of LPL. For example, the HindIII polymorphism of LPL is substantially associated with a risk of IHD. For polymorphism Ser447X, investigators have discovered that only the XX genotype significantly correlates with IHD risk. Polymorphism PvuII did not manifest a significant link with the risk of IHD. Thus, LPL polymorphism HindIII may serve as a possible risk biomarker of IHD [80].

## 14. Apolipoprotein E (ApoE)

Defects in ApoE lead to familial dysbetalipoproteinemia, also known as type III hyperlipoproteinemia, where elevated levels of cholesterol and TGs in blood plasma are caused by disrupted clearance of chylomicrons, very LDL, and LDL. Later, ApoE was studied regarding its participation in several biological processes not directly related to the transport of lipoproteins, e.g., Alzheimer’s disease, immunoregulation, and cognitive functions. Isoform 4 of ApoE, encoded by an allele of APOE, has been implicated in the elevated level of calcium ions and apoptosis after mechanical damage [81]. Data on the role of ApoE in the development of IHD are rather inconsistent. For instance, a large (~92,000 subjects) populational study on the link between ApoE and IHD clearly indicated that circulating ApoE raised the risk of IHD [82]. Sofat et al. made similar findings in their meta-analysis. It is reported that there is no evidence of a relation between ApoE blood concentration and CVD events. The validated link of an APOE genotype with CVD events may be explained by functions that are specific for isoforms and by other mechanisms rather than the concentrations of ApoE circulating with the blood [83]. By contrast, in their study, Corsetti et al. concluded that the levels of ApoE predicted CVD in females with high levels of HDL cholesterol and CRP [51]. In the elderly, high levels of ApoE in the blood precede upregulation of circulating CRP and strongly correlate with cardiovascular mortality, regardless of the APOE genotype and blood lipids.

## 15. Complement System

The complement system performs an important function in the pathogenesis of IHD and of heart failure [84,85]. The lectin pathway has a more prominent role in the induction of complement activation than do the classic and alternative pathways during ischemia-reperfusion injury [86]. Trendelenburg et al. has found that a serum level of mannose-binding lectin (MBL) is associated with lower mortality in patients with AMI who underwent transcutaneous transluminal coronary angioplasty [87]. Conversely, a low concentration of serine proteases associated with MBL is apparently a bad sign. For instance, Zhang et al. reported that the concentration of MBL-bound serine protease 2 was lower in the peripheral blood of patients after AMI, in comparison with a control group [88]. Taken together, these observations suggest that lower levels of MBL are a good sign because of a lower probability of lectin pathway activation. Several research papers have shown that an elevated level of C3 in serum and/or a higher C3/C4 ratio correlate with a higher risk of IHD. An elevated level of C4 in plasma is reported to be associated with a higher incidence of coronary events. Gombos et al. has demonstrated that higher levels of anaphylatoxin C3a in the plasma of patients with a left ventricular ejection fraction below 45% predict repeated hospitalization, cardiovascular events, and mortality [89].

## 16. Plasminogen Activator Inhibitor 1 (PAI-1)

It is reported that elevated levels of PAI1 are typically related to repeated myocardial infarction and IHD. Nonetheless, at present, the links among PAI1, early atherosclerosis, and IHD remain unclear. In several studies, it has been shown that PAI1 correlates with many traditional risk factors of IHD, such as obesity, hyperglycemia, T2DM [90], metabolic syndrome [91], and vessel wall thickness. Besides, higher expression of PAI1 is observed in coronary artery tissues in the presence of atherogenic lesions. Association of elevated levels of PAI1 in blood plasma with the incidence of IHD is noted in several prospective studies [92,93]. Nevertheless, this link has not always persisted after adjustment for cardiovascular risk factors. These discrepancies may have something to do with small sample sizes and/or limitations of the studies (e.g., only patients with T2DM, only obese people, or patients with HIV infection) [94].

## 17. Visfatin

There is evidence that the effects of visfatin on the accumulation of lipid depots are implemented by insulin receptors. By binding to these receptors, visfatin activates them. Administration of recombinant visfatin to mice acts on insulin receptor just as insulin does. The level of visfatin in circulating blood cells directly correlates with the body mass index, waist circumference, and the insulin resistance index. It is thought that visfatin participates in atherogenesis and in the pathogenesis of arterial hypertension in the presence of obesity and vascular complications of T2DM. Although further research will clarify the mechanisms behind the many well-studied physiological changes, it is already obvious that visfatin is a crucial immunoregulator with well-pronounced anti-inflammatory properties. A meta-analysis by Yu et al. [95], including 15 papers with 1053 cases of IHD and 714 control patients, suggests that, overall, visfatin concentration in peripheral blood is much higher in IHD cases than in controls. Group and meta-regression analyses revealed that the possible reasons for the heterogeneity are age, body mass index, race, diabetes mellitus, systolic arterial pressure, TGs, HDL cholesterol, and LDL cholesterol. These findings clearly show that an increase in the visfatin concentration in peripheral blood may be a risk marker of IHD. A study by Auguet et al. has revealed that the levels of visfatin are substantially higher in the secretome of the unstable atherosclerotic plaque from a carotid artery than in the secretome of a nonatherosclerotic thoracic artery. There were no differences in other analyzed adipo-/cytokines [96]. Of note, in a study by Zheng et al. on the level of visfatin in patients with T2DM, the patients were subdivided into two groups by the presence of atherosclerotic plaques. Serum levels of visfatin were higher in the group with atherosclerotic plaques. In people with atherosclerotic plaques in the carotid artery, the level of visfatin was higher than that in patients with/without plaques in the femoral artery. Pearson’s correlation analyses suggested that the serum levels of visfatin positively correlated with waist circumference, waist hip index, TGs, and the number of plaques. Logistic regression analysis revealed that a higher level of visfatin in serum is an independent predictor of the presence of atherosclerotic plaques [97].

## 18. Angiotensin II

Numerous components of the renin–angiotensin system directly affect the physiology of adipocytes, and genetic animal models have provided a wealth of information about the mechanisms underlying these effects. In general, these studies indicate that angiotensin II in visceral adipose tissue promotes accumulation of energy. For example, transgenic activation of angiotensin in the adipose tissue of mice increases obesity [98], whereas its conditional knockout in adipocytes does not affect either body weight or obesity but reduces inflammation in adipose tissue, raises metabolic activity, improves glucose tolerance, and lowers the predisposition to hypertension associated with obesity [99,100]. It is assumed that elevated levels of angiotensin in adipose tissue are sufficient for an increase in the adipose tissue volume, and that they are necessary for the inflammation associated with obesity as well as for the development of glucose tolerance impairment and hypertension. As for subtype 1 receptor of angiotensin II (AT1R), which may be involved in these phenomena, the following has been reported. Mice lacking AT1R in the whole body are resistant to diet-induced obesity, show smaller adipocytes, and do not show changes in adipocyte differentiation, indicating a possible role of AT1R in the growth of adipocytes, which manifests itself during diet-induced obesity.

## 19. Apelin

Apelin is considered a cardioprotective factor because it has effects opposite to those of the renin–angiotensin system. Apelin is expressed in several organs, including the hypothalamus, vascular endothelium, heart, lungs, and kidneys, as well as adipose tissue and the gastrointestinal tract. Its receptor APJ is widely expressed in endothelia, smooth muscles, and myocytes. In the systemic circulation, apelin causes NO-dependent vasodilatation, prevents vasoconstriction caused by angiotensin II, and exerts positive ionotropic and cardioprotective effects. Plasma levels of apelin are substantially lower in patients with atrial fibrillation than in healthy subjects. There are data on a higher risk of repeated atrial fibrillation in subjects with a lower level of apelin [101,102]. The level of apelin starts to decline early after AMI. Several days later, its level starts to grow but remains lowered until 24 weeks after AMI. This downregulation does not depend on the degree of ventricular dysfunction and prognosis [103,104]. As a rule, levels of apelin are lower in patients with IHD. In patients with unstable angina pectoris and AMI, levels of apelin are lower than those in patients with stable types of IHD. These levels also inversely correlate with the severity of coronary stenoses [105]. There is a report of a beneficial influence of apelin on reperfusion damage after AMI. There is increased expression and production of apelin in the left ventricle, whereas mRNA levels of apelin in atria are unchanged, while in blood serum, the levels of apelin are lowered, as is the expression of apelin receptor AJP.

## 20. Omentin

Expression of the omentin gene and the omentin serum levels are lower in obese people and inversely correlate with the body mass index, waist circumference, insulin resistance, and IHD. Conversely, there is a positive correlation of omentin with serum adiponectin and HDL cholesterol. Furthermore, omentin increases insulin-induced glucose reuptake and participates in the regulation of insulin sensitivity and, therefore, may exert a protective action against the worsening of insulin resistance. Regarding the influence of omentin on the cardiovascular system, as mentioned above, lower levels of omentin are seen in patients with IHD [106,107]. In case of heart failure, the levels of omentin are significantly lower in people who experienced a greater number of cardiac events in the long term (death, repeated hospitalization) and in patients with more severe symptoms (with New York Heart Association (NYHA) class IV in comparison with NYHA II and III) [108].

## 21. Monocytic Chemoattractant Protein-1 (MCP-1)

Induction of chemokines is a characteristic feature of the inflammatory response associated with ischemia-reperfusion injury in many tissues. Analysis of biopsies from patients and animal models by hybridization and immunostaining in situ have revealed mRNA and protein expression of MCP1 in an ischemic myocardium. Elevated levels of MCP1 in blood serum have been detected in patients with IHD and were implicated in the risk of myocardial infarction and left ventricle dysfunction [109,110]. Quantitation of MCP1 in the coronary blood of patients with tension-related unstable angina pectoris has shown a link between the levels of MCP1 and the degree of coronary atherosclerosis as evidenced by coronary angiography. In the OPUS-TIMI 16 study, levels of MCP1 above the 75th percentile were associated with a higher risk of death or AMI after 10 months, even after adjustment for the traditional risk factors. Furthermore, according to Lee et al., the levels of circulating MCP1 positively correlate with a greater amount of visceral adipose tissue and IHD with multiple vascular damage [109]. Even though MCP1 is a promising biomarker, further research is needed to determine its clinical value.

## 22. Retinol-Binding Protein 4 (RBP4)

Upregulation of retinol-binding protein 4 (RBP4) in human blood serum is linked with insulin resistance, the development of T2DM, and such clinical manifestations of metabolic syndrome as obesity, glucose intolerance, dyslipidemia, and hypertension [111,112]. It has been demonstrated that patients with atherosclerosis of carotid arteries in combination with IHD have higher levels of RBP4 [113]. Liu et al. has noted that higher levels of RBP4 and adiponectin are related to higher rates of death from CVDs among males with T2DM [114]. Similar results have been obtained by Ingelsson et al. In the elderly, RBP4 concentrations correlate with metabolic syndrome and its components both in males and females as well as with a history of cerebrovascular disease in males [115]. In a study on a female population, Alkharfy et al. found that serum RBP4 concentration correlated with various well-established risk factors of CVDs; accordingly, they proposed that RBP4 may serve as an independent predictor of CVDs in females [116]. All the above data are consistent with the hypothesis that circulating RBP4 is a marker of metabolic complications, and possibly of atherosclerosis too.

## 23. Molecular and Genetic Studies

Recent years have witnessed a number of molecular genetic studies on the molecules of the secretory activity of visceral adipocytes, again, mostly in relation to pathologies of the cardiovascular and endocrine systems.

There has been research into the expression of adiponectin genes in patients with various types of cardiovascular pathologies [117,118]. One research group studied the mRNA expression of adiponectin in adipose tissue samples from 11 males after coronary artery bypass grafting and 10 males with a replaced heart valve. It was found that the mRNA expression was high in abdominal adipose tissue [119]. There is a report of a lower level of adiponectin with a concurrent increase in the expression of CD48 and MCP1 in the adipose tissue of patients with T2DM [120]. Underexpression of the adiponectin gene (*ADIPOQ*) and simultaneous upregulation of leptin (*LEP*) were detected in the analysis of the transcriptome from the adipocytes of patients with metabolic syndrome after coronary artery bypass grafting [121]. Other researchers have revealed that adiponectin expression is lower in patients with hypertension [122] or with IHD [123,124]. Another study has uncovered an insignificant increase in the expression of this gene in patients with IHD [17]. During induction of adipogenesis in the samples of visceral adipocytes from patients with IHD after a surgical intervention, investigators noted upregulation of *ADIPOQ* in response to various concentrations of glucose [125].

## 24. Conclusions and Future Perspectives

Thus, there is large number of biomarkers secreted by visceral adipocytes. All of them can substantially affect the development of atherosclerosis, vascular inflammation, and, as a consequence, stroke, myocardial infarction, and other cardiovascular events [126]. Nonetheless, the effects of most biomarkers discovered to date are usually either weak, poorly studied, or inconsistent and require further research.

It should be noted that the evidence accumulated so far regarding the secretory function of adipose tissue and its involvement in the pathogenesis of some socially significant diseases (such as obesity, T2DM, and atherosclerosis) suggests that it is important to continue studying the molecular basis of this secretory function. Today, there are virtually no data on the secretory function of adipose tissue and its role in the pathogenesis of various socially significant diseases and pathologies (not only cardiovascular and endocrine) in young adults at employable and reproductive age. These future studies seem to be especially relevant because they include medical examination of young adults living in strongly continental and subarctic climatic geographic conditions.

Overall, studies on the molecular genetic factors underlying adipose tissue function in the context of the above conditions may lead to a deeper understanding of the etiopathogenesis of not only cardiovascular but also other common socially significant diseases and pathologies in young adults as well as help to develop an effective strategy for their prophylaxis and management.
